# Protective Effect of Onion Extract on Bleomycin-Induced Cytotoxicity and Genotoxicity in Human Lymphocytes

**DOI:** 10.3390/ijerph13020227

**Published:** 2016-02-22

**Authors:** Yoon Hee Cho, Joong Won Lee, Hae Dong Woo, Sunyeong Lee, Yang Jee Kim, Younghyun Lee, Sangah Shin, Hyojee Joung, Hai Won Chung

**Affiliations:** 1 School of Public Health, Seoul National University, Gwanak-gu, Seoul 151-742, Korea; lasthitter@gmail.com (J.W.L.); ssun1987@snu.ac.kr (S.L.); ychi@snu.ac.kr (Y.L.); hjjoung@snu.ac.kr (H.J.); chunghw@snu.ac.kr (H.W.C.); 2 Department of Biomedical and Pharmaceutical Sciences, College of Health Professions and Biomedical Sciences, University of Montana, 32 Campus Drive, Missoula, MT 59812, USA; 3 Molecular Epidemiology Branch, Division of Cancer Epidemiology and Prevention, Research Institute, National Cancer Center, Ilsandong-gu, Gyeonggi-do 410-769, Korea; eastsea93@hanmail.net; 4 Da Vinci College of General Education, Chung-Ang University, Dongjak-Gu, Seoul 156-756, Korea; yangjee4@hanmail.net; 5 Institute of Health and Environment, Seoul National University, Gwanak-gu, Seoul 151-742, Korea; ssa8320@snu.ac.kr

**Keywords:** radiation, onion extract, bleomycin, cytokinesis-blocked micronucleus assay, single cell gel electrophoresis assay

## Abstract

Following one of the world’s largest nuclear accidents, occured at Fukushima, Japan in 2011, a significant scientific effort has focused on minimizing the potential adverse health effects due to radiation exposure. The use of natural dietary antioxidants to reduce the risk of radiation-induced oxidative DNA damage is a simple strategy for minimizing radiation-related cancer rates and improving overall health. The onion is among the richest sources of dietary flavonoids and is an important food for increasing their overall intake. Therefore, we examined the effect of an onion extract on cyto- and geno-toxicity in human lymphocytes treated with bleomycin (BLM), a radiomimetic agent. In addition, we measured the frequency of micronuclei (MN) and DNA damage following treatment with BLM using a cytokinesis-blocked micronucleus assay and a single cell gel electrophoresis assay. We observed a significant increase in cell viability in lymphocytes treated with onion extract then exposed to BLM compared to cells treated with BLM alone. The frequency of BLM induced MN and DNA damage increased in a dose-dependent manner; however, when lymphocytes were pretreated with onion extract (10 and 20 μL/mL), the frequency of BLM-induced MN was decreased at all doses of BLM and DNA damage was decreased at 3 μg/mL of BLM. These results suggest that onion extract may have protective effects against BLM-induced cyto- and genotoxicity in human lymphocytes.

## 1. Introduction

After one of the world’s largest nuclear accidents at the Fukushima Daiichi Nuclear Power Plant, caused by the East Japan Earthquake of 2011, the potential adverse health effects of radiation exposure have become a major concern worldwide [1]. Consequently, scientific efforts have focused on ways to minimize the adverse health effects from radiation exposure. Radiation is a well-known human carcinogen that can induce a large variety of biological responses depending on dose, duration, dose rate, and type of the radiation. There are two broad types of radiation, ionizing and non-ionizing. Ionizing radiation (IR) is high-frequency radiation that has extremely high energy that can break off electrons from (ionize) an atom. IR is also known to produce reactive oxygen species (ROS), which result in an increase in oxidative stress and can lead to genetic damage that may contribute to the early stages of cancer progression [2,3].

Bleomycin (BLM) is considered a radiomimetic agent because of the similarities between its genotoxicity profile and that of IR [4]. Most notably, both IR and BLM are S-phase-independent, inducing chromosomal damage at all stages of the cell cycle [5,6]. BLM induces DNA strand breaks in the presence of iron and oxygen, resulting in production of DNA adducts and excess ROS leading to oxidative stress, mitochondrial leakage, and apoptosis [7].

Diets high in fruits and vegetables are known to be protective against a variety of diseases. Particularly, antioxidants are thought to be the principal nutrients responsible for the protective effects of fruits and vegetables [8]. There is extensive literature on the antioxidant effects of food-derived flavonoids such as quercetin, kaempferol and myricetin. Flavonoids are reported to inhibit the activity of various enzyme systems, such as cyclooxygenase and lipoxygenase, through their antioxidant properties, free radical scavenging ability, and chelation of divalent cations [9,10]. Several flavonoids have also been reported to have anti-mutagenic properties against various mutagens and carcinogens [11,12,13]. Since dietary inhibitors of mutagenesis and carcinogenesis may be helpful for human cancer prevention and overall improvement in health, they are of particular interest [14].

Onions (*Allium cepa* L.) are one of the most widely consumed vegetables worldwide. Since onions contain large amounts of flavonoids and constitute one of the major sources of flavonoids in the human diet [15], they have been suggested as a resource for dietary antioxidants [16]. Onions are also known to possess scavenging properties against reactive oxidative species [17].

The use of natural dietary antioxidants, particularly flavonoids, to reduce the risk of radiation-induced oxidative DNA damage might be a simple method for reducing radiation-related cancer and improving overall health [18]. There are limited studies on the effects of diet in radiation-exposed populations; therefore, it is important to investigate the protective effects of flavonoids on chromosomal damage following exposure to IR. To this end, the aim of the present study was to determine the protective effects of onion on radiation-induced genetic damage. We examined cyto- and geno-toxic effects induced by BLM, a radiomimetic agent and ROS generator, in the presence or absence of onion extract in human lymphocytes. The single-cell gel electrophoresis and cytokinesis-block micronucleus assays (CBMN) represent reliable tests to measure genetic damage and were therefore used to measure DNA and chromosomal damage in these experiments [19,20].

## 2. Experimental Section

### 2.1. Cell Isolation and Culture

Peripheral blood was obtained from one consenting healthy female donor aged 28. The donor was a non-smoker at the time of blood draw. Separation of peripheral blood mononuclear cells (PBMCs) was carried out under sterile conditions on Ficoll-Paque gradients by the method of Boyum [21]. In brief, the PBMCs were isolated by buoyant density centrifugation at 400 ×*g* using Ficoll-Paque™ (Amersham Biosciences, Uppsala, Sweden), removed from the interface, washed twice, and resuspended in RPMI 1640 (Gibco-Invitrogen, Carlsbad, CA, USA) that was supplemented with 10% fetal bovine serum (Gibco) and 100 U/mL penicillin/streptomycin (Sigma-Aldrich, St. Louis, MO, USA). The cultures were stimulated with 1% phytohemagglutinin (PHA, Gibco). The PBMCs were cultured in 5% CO_2_ at 37 °C.

### 2.2. Preparation of Onion Extracts

Fresh onions (*Allium cepa* L., Turbo) were purchased from a local market. The onions were washed with sterile distilled water and allowed to air dry for one hour. The outer covering of the onions were manually removed and the peeled onions were then placed into alcohol (60%) and extracted for 12 h at stable pressure (1–2 kg/cm^2^) and temperature (85 °C–90 °C). Prior to treatment, the onion extracts were filtered using filter paper (Advanced Toyo, Tokyo, Japan).

### 2.3. Cell Treatment

The concentrated onion extract was added to the PBMCs with a single treatment in an amount that is the equivalent in ratio to a 50 kg adult eating 750 g or 1500 g of onion. Thus, the cells were treated with onion extracts at 10 and 20 μL/mL, and incubated for 1 h. Then 1 and 3 μg/mL of BLM (CAS No: 9041-93-4, Sigma-Aldrich) were added to the culture and incubated for an additional 3 h. The onion extract and BLM treated cells were washed with phosphate-buffered saline (PBS) twice and onion extracts and PHA were reapplied to the cells a second time.

### 2.4. Cell Viability Assay

The PBMCs were grown at a density of 1 × 10^6^ cells/mL for the trypan blue exclusion assay [22]. Cells were stimulated with PHA for 24 h and then exposed to the onion extract (20 μL/mL) and BLM (1 μg/mL) for 0, 24, 48 and 72 h. Cell numbers were determined using a Neubauer hemocytometer and viable cells were analyzed by determining their ability to exclude the dye. The cytoplasm of a viable cell is clear, whereas a non-viable cell has a blue cytoplasm. We calculated cell viability (%) as follows: Cell viability (%) = [total number of viable cells (trypan blue-negative) cells]/[total number of cells including trypan blue-positive and negative cells] × 100.

### 2.5. Cytokinesis Block Micronucleus Assay

The cytokinesis block micronucleus assay was performed as described by Fenech [23]. The onion extract and BLM treated cell cultures were incubated for 44 h and cytochalasin-B (CAS No: 14930-96-2, 4 μg/mL, Sigma-Aldrich) was added for an addtional 28 h. Exponentially growing cells were collected and subsequently swollen in a hypotonic 0.075M KCl solution for 3 minutes at 37 °C and then twice resuspended in a 3:1 mixture of methanol to acetic acid. Air-dried preparations were made and stained by Giemsa staining. We scored 2000 binucleated (BN) cells from each slide, and micronuclei (MN) were identified according to the criteria of Fenech [19]. All slides were coded and scored blindly. The frequency of MN is expressed as total number of MN *per* 2000 BN cells.

### 2.6. Single-Cell Gel Electrophoresis (Comet Assay)

The alkaline comet assay was performed as described by Singh *et al.*, [24] to assess DNA strand breaks. Briefly, PBMCs were stimulated with PHA for 24 h then treated with the onion extract for 1 hour followed by addition of BLM and further incubation for 3 h. After washing twice with PBS, the cells were maintained at 4 °C to prevent DNA repair. The cells were mixed with 85 μL of 0.5% low-melting agarose (Sigma-Aldrich) and placed on a microscope slide that was pre-coated with 0.6% normal melting agarose (Sigma-Aldrich). Another 85 μL of low-melting agarose was added to form the top layer. The cells were lysed at 4 °C in an ice-cold, freshly prepared solution of 2.5M NaCl, 100 mN Na_2_EDTA, 10 mM Tris-HCl, 1% Triton X-100, and 10% dimethyl sulfoxide at pH 10. The slides were placed on a horizontal gel electrophoresis unit. DNA was allowed to unwind for 20 min in an electrophoretic alkaline buffer (1 mM Na_2_EDTA, 300 mM NaOH, pH 13) and electrophoresed in the same buffer for 25 min at 0.78 V/cm and 330 mA. Once electrophoresis was complete, the slides were gently washed in a neutralization buffer (0.4 M Tris-HCl, pH 7.5). Subsequently, the slides were dried and fixed with absolute ethanol for at least 60 s. Immediately before examination, the slides were stained with 100 μL ethidium bromide (10 μL/mL, Sigma-Aldrich). For visualization of DNA damage, observations were made using the 40× objective of a fluorescent microscope (Nikon, Tokyo, Japan) that was equipped with an excitation filter of 515–560 nm and a barrier filter of 590 nm. Images of 60 randomly selected cells from each sample were examined using the KOMET5.5 image analysis system (Andor Technology, Belfast, UK) [20,25]. The Olive tail moment (the comet tail length and the DNA content of the tail), defined as the fraction of total DNA in the tail, was used as an index of damage [26,27].

### 2.7. Quantitative Analysis of Quercetin by High Performance Liquid Chromatography (HPLC)

LC-MS/MS analysis was performed by triple quadrupole mass spectrometry coupled with an HPLC system (Thermo Scientific, San Jose, CA, USA) to determine the concentration of quercetin, the major flavonoid in onion extract. Quercetin was separated on a Zorbax Extend-C18 column (2.1 × 150 mm, 5 μm, Agilent Technologies, Santa Clara, CA, USA). The mobile phase consisted of 0.1% formic acid in water (A) and 0.1% formic acid in acetonitrile (B) with a flow rate of 0.2 mL/min. The gradient conditions were set as follows: 10%–50% B for 10 min, isocratic 50% B for 2 min and 10% B for 7 min to equilibrate the column. Injection volume was 10 μL. The concentrations used for the standard curve were 0.1, 0.2, and 0.5 μM. Quercetin was monitored in the negative ion mode with electrospray ionization. The optimum MS conditions were as follows: spray voltage 3000, capillary temperature 300 °C, and sheath gas pressure and aux gas pressure of 40 (psi) and 10 (Arb), respectively. Quantitative analysis of quercetin was performed using selective reaction monitoring mode (m/z 301/150.980). Data processing was performed using the Xcalibur software (Thermo Scientific).

### 2.8. Statistical Analysis

Statistical analysis was performed using the SPSS 10.0 for Windows statistical package (SPSS Inc., Chicago, IL, USA). All experiments were performed in triplicate, and the inter-group difference among three experiments was tested by analysis of variance (ANOVA). The association of abnormal yields with BLM doses was tested by the Kendall rank correlation coefficient (τ). The variations in cell viability, MN frequency, and cells with DNA damage among different treatment groups were tested with the Mann-Whitney and Wilcoxon tests. Data are expressed as means ± SD or SEM of three different experiments, and *p* < 0.05 was considered statistically significant.

## 3. Results

### 3.1. Cell Viability with Onion Extracts and BLM Treatment

**Figure 1:**
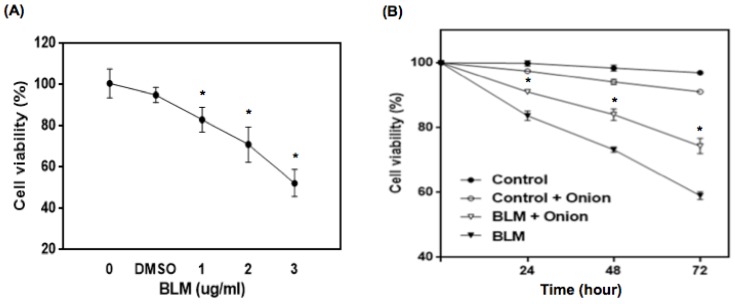
Influence of BLM on the viability of human PBMCs as measured by the trypan blue exclusion method. (**A**) Human PBMCs were treated with the indicated concentrations of BLM for 24 hours. A significant decrease in the number of trypan blue-negative cells was evident in the BLM-treated lymphocytes. * *p* < 0.01 compared to untreated controls; (**B**) Lymphocytes were treated with or without 20 μL/mL of onion extract and 1μg/mL of BLM. A significant increase in the number of trypan blue-negative cells was evident in the cells treated with onion extract compared to the cells treated with BLM only. The data shown represent means ± SD of three different experiments. * *p* < 0.05 compared to the cells treated with BLM only.

The viability of PBMCs treated with various concentrations of BLM (1, 2, and 3 μg/mL) was determined using a Trypan blue-exclusion assay. BLM-treated cells exhibited significantly decreased viability compared to the controls and/or DMSO treated cells after 24 hours. BLM treatment decreased the number of viable cells in a dose-dependent manner as indicated by the number of trypan blue-positive cells (*p* < 0.001; Figure 1A). In Figure 1B, there was a significant decrease in viability of onion extract (20 μL/mL) treated cells, compared to the control cells after 72 h (*p* < 0.05), suggesting the onion extract at a high dose caused cytotoxicity. After treatment of 1 μg/mL of BLM to the PBMCs, there was a significant decrease in the number of trypan blue-negative cells at all time points examined compared to the control cells (*p* < 0.01). In contrast, supplementing BLM-treated cells with onion extract (20 μL/mL) increased the number of trypan blue-negative cells compared to cells treated with BLM alone (*p* < 0.05).

### 3.2. Protective Effect of Onion Extracts on the Frequency of BLM-Induced MN

The CBMN assay was used to determine the effect of onion extract on BLM-induced genotoxicity. The micronucleated cytokinesis-blocked (MNCB) cells and MN frequencies were significantly increased after BLM exposure in a dose-dependent manner as shown in Table 1 (*p* < 0.001). The total MN frequencies in control was 18 ± 2.65, and increased to 36 ± 6.24 and 60 ± 4.36 after 1 and 3 μg/mL of BLM, respectively. The frequency of MN induced by 1 μg/mL of BLM exposure was significantly reduced by pretreating the cells with 20 μL/mL of onion extract, while 3 μg/mL of BLM-induced MN was significantly reduced by both 10 and 20 μL/mL of onion extract (*p* < 0.05).

### 3.3. Protective Effect of the Onion Extract on BLM-Induced DNA Damage

**Figure 2:**
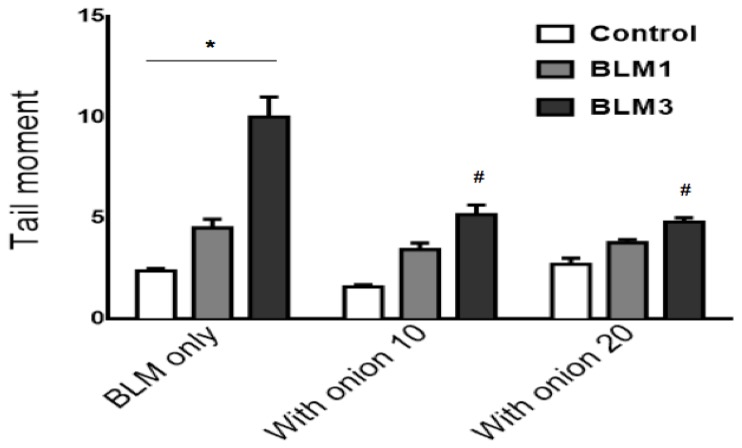
Protective effect of onion extract on BLM-induced DNA damage determined by single cell gel electrophoresis in human PBMCs. The DNA damage significantly increased with BLM doses (BLM 1 and 3 = 1 and 3 μg/mL of bleomycin, respectivley). A significant decrease in DNA damage was observed in the cells treated with 10 or 20 μL/mL of onion extract compared to the cells treated with BLM 3 only. Data represent means ± SEM of three different experiments * Significant increase with bleomycin doses (*p* < 0.001); ^#^ Significantly different from BLM 3 exposure only (*p* < 0.05).

The comet assay, a conventional measure of DNA damage, was used to determine DNA single-strand breaks induced by BLM with or without onion extract. Figure 2 shows that the tail moment of the cells significantly increased with BLM dosage (*p* < 0.001). Pretreatment of cells with both 10 and 20 μL/mL of onion extract significantly decreased the DNA damage induced by 3 μg/mL of BLM (*p* < 0.05).

### 3.4. Quantitative Analysis of Quercetin

HPLC analysis confirmed the presence of quercetin in onion extracts with a concentration of 17.1 μM.

## 4. Discussion

After the Fukushima tragedy, the potential adverse health effects of radiation exposure became a major worldwide concern. The World Health Organization (WHO) is currently developing a health risk assessment to provide a preliminary evaluation of radiation exposure [28]. In particular, the WHO has conducted studies to estimate the doses of radiation exposure received by residents in the area around the Fukushima plant, studies on the management of radioactive waste, studies to improve the health care program/plans for Fukushima residents, and studies to minimize the adverse health effects of radiation exposure [29,30,31,32,33].

Currently, there is a great deal of interest in the use of dietary interventions to assist in protecting the public’s health from chronic radiation exposure. Specifically, dietary inhibitors of mutagenesis and carcinogenesis may be helpful for preventing human cancer and improving overall health [11]. It has been well described that IR induces DNA damage through direct ionization of DNA or through free ROS formed by radiolysis of water [34]. ROS produced by IR result in an increase in oxidative stress, leading to the formation of DNA-reactive products that could induce mutations in target tissues [35]. The biological and genotoxic effects of IR exposure and its relationship to carcinogenesis have received much attention in the past decade. The use of natural dietary antioxidants to reduce the risk of radiation-induced oxidative DNA damage might be a simple and effective way for preventing radiation-related cancers. Therefore, we evaluated the potential protective effects of an onion extract on BLM-induced cyto- and genotoxicity in human lymphocytes. Since onions contain large amounts of flavonoids and phenolic compounds, they have been suggested as a major resource for increasing dietary antioxidant intake [36]. Onions also have ROS scavenging activity, but there is very little information on the protective effects of onion extract against radiation or BLM-induced DNA damage.

The results of this study demonstrate that pretreatment of normal human PBMCs with an onion extract results in a significant increase in cell survival upon exposure to BLM-suggesting that the onion possesses significant activity as a cyto-protective agent. We determined the protect effects of onion extract on BLM-induced genotoxicity in lymphocytes by using CBMN and single-cell gel electrophoresis assays. Data presented in Table 1 and Figure 2 show a significant decrease in frequency of MN and DNA damage in cells treated with onion compared to cells treated with BLM only. The cyto- and genotoxicity of BLM is thought to be mainly due to excessive production of ROS. Therefore, pretreating cells with onion extract may reduce BLM-induced oxidative stress through the radical scavenging activity of the onion extract constituents. Concordantly, the literature has shown very strong evidence that onion extract can protect against a wide variety of ROS generating agents [37]. Alpsoy *et al*., recently showed that onion extracts have a protective effect on cadmium-induced oxidative stress [38]. Similarly, Mete *et al*., reported that pretreatment with onion extract protects against doxorubicin-induced hepatotoxicity due to its antioxidant properties [39]. In addition, previous studies have shown that flavonoids afford cyto-protective effects against micronucleus induction in irradiated mice and lymphocytes [40,41]. Similar to our results, Heo *et al*., reported anti-clastogenic effects of flavonoid derivatives against induction of chromosomal aberrations following BLM exposure [42]. Furthermore, studies suggest that the free radical scavenging activity of dietary flavonoids may be due to additional factors, such as stimulation of glutathione and other antioxidant molecules [43,44].

The antioxidant effect of onion extract is mostly due to phenolic acids and flavonoids, which constitute the most predominant functional components [45]. These compounds have antioxidant activity and can quench superoxide anions [46,47]. In particular, distinct structural components contribute to their strong scavenging activity against superoxide anions, including hydroxylation of the C3 position, increased numbers of hydroxyl group in ring B, and a saturated C2–C3 bond [46,47]. Inal and Kahraman reported that flavonoids such as quercetin prevent a decrease in superoxide dismutase activity by trapping superoxide radicals [48]. Furthermore, flavonoids have a strong affinity for iron ions, which are known to catalyze many processes leading to the formation of free radicals [49]. As such, the ability of flavonoids to chelate metal ions and inhibit lipid peroxidation may act in synergy toward scavenging free radicals [50]. In support of this, Magnani *et al*., showed that flavonoids can scavenge free radicals and chelate iron, thereby preventing hydroxyl radical-promoting Fenton reactions [47]. The protective effects of flavonoids against cyto- and geno-toxicity of BLM can be also explained by other mechanisms such as DNA repair. Phenolic compounds are good electron or H atom donors; therefore, they may repair some oxidative DNA damage [51]. A study by Charles *et al*., [52] reported that some flavonoids activate non-homologous end-joining to increase DNA double-strand break repair. Similarly, the same group demonstrated that flavonoids modulate alkylated DNA repair kinetics [53]. However, the mechanisms underlying this DNA repair remain unclear and further mechanistic work is needed to draw conclusions.

Extraction of flavonoids using water is known to be inefficient since the solubility of flavonoids in water at ambient conditions is low [54]. Therefore, the conventional way to extract flavonoids from plant material is with organic solvents such as methanol, ethanol or ethyl acetate [55]. However, a comparison of the efficiency of flavonoid extraction using different solvents is difficult because various solvent systems and extraction conditions have been employed in other studies. We prepared the onion extract in ethanol since ethanol has been shown to be the most cost effective and efficient solvent [56,57].

Onions contain various flavonoids such as quercetin, ferulic acid, gallic acid, protocatechuic acid, and kaempferol, which contribute to the radical scavenging and metal ion chelating properties and result in significant anti-genotoxic and protective effects [17]. However, the bioavailability of these diet flavonoids can vary according to their chemical structure and food sources. The bioavailability of quercetin is very high, whereas other flavonoids are relatively low [58,59,60,61]. Moreover, quercetin in particular is the major component in onions, and it has shown much promise as an antioxidant agent [62,63,64]. Therefore, we determined the concentration of quercetin only in our samples using HPLC. Consequently, lack of information regarding the whole contents of onion extract is a major limitation of this study.

An epidemiology study measuring cancer risk among a group of atomic bomb survivors showed daily intake of vegetables and fruit reduced the risk of radiation-associated cancer [33]. However, other studies showed no association between fruit or vegetable consumption and cancer risk after radiation exposure among atomic bomb survivors [31,65]. Studies examining the effect of diet on radiation-exposed populations are limited and controversial. Therefore, little is known regarding the impact of a diet high in fruits and vegetables with respect to radiation-induced cancer. Examination of the protective effects of onion extracts against BLM exposure rather than irradiation exposure is another limitation, although BLM acts as a radiomimetic agent. Thus, further study with irradiated cells to determine the direct effects of onion extract against radiation exposure will be needed. In addition, we treated cells with a high dose of onion extract in order to ensure a significant protective effect against BLM-induced toxicity with a single treatment in an *in vitro* experiment. Therefore, caution is required when extrapolating this data to estimate the daily onion intake required for prevention of BLM-induced toxicity *in vivo*. Despite these limitations, our study provides useful information. To our knowledge, this is the first study to evaluate the anti-genotoxic and protective effects of onion extract against BLM-induced DNA damage in human lymphocytes *in vitro*.

## 5. Conclusions

The present study demonstrates protective effects of onion extract against the cytotoxicity and genotoxicity of BLM, a radiomimetic agent. Here for the first time, we provide confirmatory evidence for nutritional protection, particularly by onion extract containing abundant flavonoids, against DNA damage induced by a radiomimetic agent in human lymphocytes. Our results may provide useful information that can be used to set up strategies to minimize radiation-induced health effects.

## Figures and Tables

**Table 1:** The effect of onion extract on BLM-induced micronuclei frequency in human lymphocytes.

Treatment	No. of MNCB ^a^ Cells/2000 BN Cells	Multi MNCB ^b^/MNCB Cells (%) ^†^	Total No. of MN/2000 BN Cells
Control	17 ± 2.65	5.99 ± 0.01	18 ± 2.65
Control + onion 10 ^c^	13 ± 2.52	7.80 ± 0.07	14 ± 3.61
Control + onion 20 ^d^	14 ± 1.73	14.9 ± 0.09	16 ± 1.00
BLM 1 ^e^	32 ± 6.56	12.8 ± 0.09	36 ± 6.24
BLM 1 + onion 10	22 ± 4.36	13.4 ± 0.03	25 ± 5.29
BLM 1 + onion 20	18 ± 2.65 ^#^	11.3 ± 0.02	20 ± 2.65 ^#^
BLM 3 ^f^	46 ± 2.65 *	27.5 ± 0.02	60 ± 4.36 *
BLM 3 + onion 10	33 ± 3.61	15.0 ± 0.02	38 ± 4.36 ^#^
BLM 3 + onion 20	29 ± 2.65 ^#^	24.0 ± 0.04	37 ± 2.65 ^#^

The data shown represent means ± SD of three different experiments; ^a^ MNCB = micronucleated cytokinesis-blocked; ^b^ Multi MNCB = cells with several micronuclei; ^c^ Onion 10 = 10 μL/mL of onion extract; ^d^ Onion 20 = 20 μL/mL of onion extract; ^e^ BLM 1 = 1 μg/mL of bleomycin; ^f^ BLM 3 = 3 μg/mL of bleomycin; ^†^ The ratios were calculated from individual values; * Significant increase with bleomycin doses (*p* < 0.001); ^#^ Significantly different from BLM exposure only (*p* < 0.05).
